# Supplementary material for: LSH-GAN enables in-silico generation of cells for small sample high dimensional scRNA-seq data
Source: Commun Biol. 2022 Jun 10;5:577. doi: 10.1038/s42003-022-03473-y (PMC9187761; doi:10.1038/s42003-022-03473-y)
Supplement: Supplementary file 2 — Supplementary Information [file 42003_2022_3473_MOESM2_ESM.pdf]

LSH-GAN enables in-silico generation of cells for small sample high dimensional scRNA-seq data

Snehalika Lall[a], Sumanta Ray[b], Sanghamitra Bandyopadhyay[a]

[a] *Machine Intelligence Unit, Indian Statistical Institute, Kolkata, West Bengal 700108, India.*
[b] *Genome Data Science, Bielefeld University, Germany*

Supplementary Note 1: Locality Sensitive Hashing

Locality Sensitive Hashing (LSH) [1, 2] is widely used in nearest neighbour searching to reduce the dimensionality of data. LSH utilizes locality-sensitive hash functions which hash similar objects into the same bucket with a high probability. The number of buckets is much lesser than the universe of possible items, thus reduces the search space of the query objects (see supplement for detailed description of LSH technique). The approximate nearest-neighbors of d query objects are searched within the bucket, which decreases the computation time from $\mathcal{O}(d^2)$ to $\mathcal{O}(d \log d)$.

In this work, We utilized python sklearn implementation of *LSHForest* module with default parameters except the *number of estimators* which is set as 30.

Supplementary Note 2: Overview of datasets

The brief description of dataset is given here. The single-cell RNA sequence datasets used for evaluation of our proposed approach are downloaded from Gene Expression Omnibus (GEO) https://www.ncbi.nlm.nih.gov/geo/.Those are discussed below.

Yan Dataset: This is a human preimplantation embryo and embryonic stem cell dataset. The average total read count in the expression matrix is 25,228,939 reads. There are 7 cell types, including labelled 4-cell, 8-cell, zygote, Late

blastocyst and 16-cell, downloaded from GEO under accession no. GSE36552 [3].

Klein Dataset: This dataset was generated by the droplet barcoding method with an average total read count of 20,033.40 reads in the expression matrix. A total of eight single cell data sets are submitted: 3 for mouse embryonic stem (ES) cells (1 biological replicate, 2 technical replicates); 3 samples following LIF withdrawal (days 2,4, 7); one pure RNA data set (from human lymphoblast K562 cells); and one sample of single K562 cells. The dataset was downloaded from GEO under accession no.GSE65525 [4].

Pollen: scRNA-seq: Strand-specific reads were aligned to the human reference genome, Ensembl GRCh37/hg19 release 75, using TopHat v2.0.10 with the flags (–library-type fr-firststrand –microexon-search). De novo transcriptome assembly was performed separately on rRNA depletion total RNA-seq alignments, and on polyA selection RNA-seq alignments, using Cufflinks v2.2.1 with the flags Dataset Libraries were generated from 600 individual cells in parallel. It contains 11 cell types. The dataset was downloaded from GEO under accession no GSM1832359 [5].

Darmanis: It contains single cell RNA sequencing on 466 cells to capture the cellular complexity of the adult and fetal human brain at a whole transcriptome level. Healthy adult temporal lobe tissue was obtained from epileptic patients during temporal lobectomy for medically refractory seizures. The dataset was downloaded from GEO under accession no GSE67835 [6].

Melanoma [7]: The dataset describes the diversity of expression states within melanoma tumors, it is obtained freshly resected samples, disaggregated the samples, sorted into single cells, and profiled them by single-cell RNA-seq. It is downloaded from GEO under accession no. GSE72056. It contains 19783 number of genes and 68579 cells with 14 cell types. Tumors were disaggregated, sorted into single cells, and profiled by Smart-seq2.

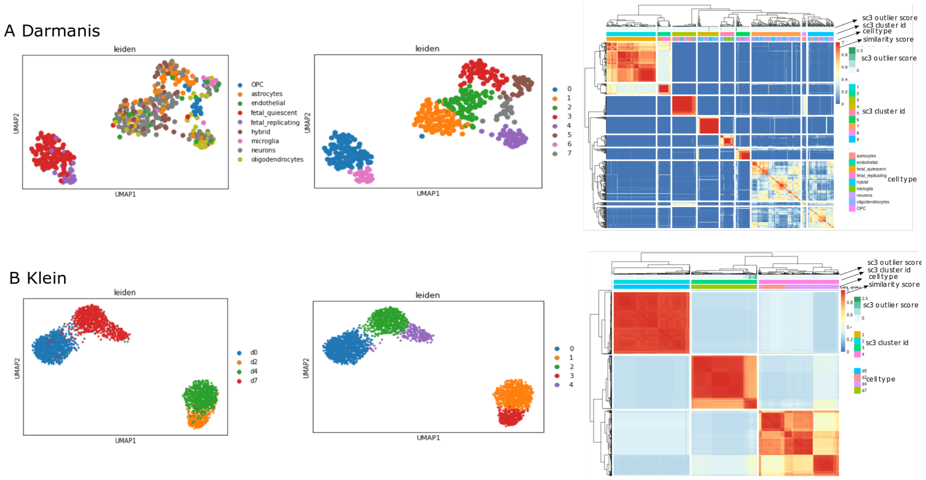

Supplementary Figure 1: Figure shows the clustering results of Darmanis and Klein data sets. Panel-A shows the t-SNE visualization of clustering results (original and predicted labels),and consensus clustering plots of obtained clusters for darmanis data. Panel-B shows the t-SNE visualization of clustering results (original and predicted labels),and consensus clustering plots of obtained clusters for Klein data.

Supplementary Note 3: Clustering results on Darmanis and Klein data

Here we provide detailed results of clustering analysis on the two datasets: Darmanis and Klein (see main text for the results on YAN and Pollen data)

Prameter selection of LSH-GAN

The number of nearest neighbor (k) and the number of iteration (t) are two main parameters of the LSH-step (see Algorithm-1 in main text), tuning of which affects the amount of sampling given to the generator for training the LSH-GAN model. We vary k and t in the range $\{5, 10, 15, 20\}$ and $\{1, 2\}$, respectively, and choose that value for which the Wasserstein distance between generated and real samples is reported to be minimum. We fixed the amount of sampling using $k = 5, t = 1$ for Pollen, Yan, Darmanis datasets and $k = 5, t = 2$ for Klein dataset and Melanoma datasets (see supplementary table 1)

Supplementary Table 1: Wasserstein distance between generated and real samples for different range of parameters k and t.

	t=1					t=2				
	Yan	Pollen	Darmanis	Melanoma	Klein	Yan	Pollen	Darmanis	Melanoma	Klein
k=5	0.23	0.21	0.29	0.36	0.32	0.24	0.28	0.35	0.31	0.3
k=10	0.32	0.3	0.39	0.43	0.5	0.29	0.28	0.35	0.37	0.39
k=15	0.29	0.33	0.4	0.46	0.48	0.37	0.38	0.41	0.38	0.4
k=20	0.3	0.37	0.41	0.5	0.5	0.43	0.44	0.48	0.4	0.42

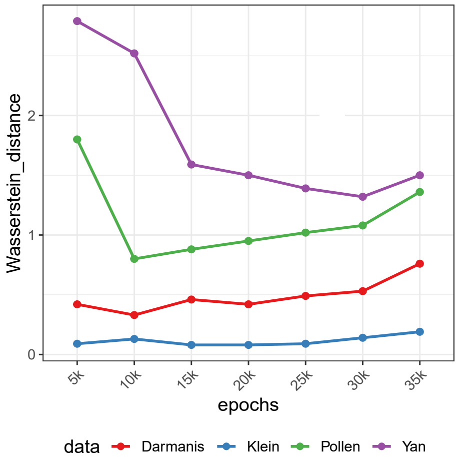

Supplementary Figure 2: Figure shows Wasserstein metric between real and generated data distribution across different epochs for scRNA-seq datasets.

We trained the LSH-GAN model in five scRNA-seq datasets: Darmanis, Yan, Pollen, Klein and Melanoma (see table-1 of main text). Here, a sub-sample of real data distribution is augmented with prior noise and used as the input to the generator network. The generated data using LSH-GAN (with k=5) is validated by computing the Wasserstein metric between the real and generated data distribution for different epochs (see supplementary figure 2). For each data, we note the epoch (e_{opt}), which results in the lowest Wasserstein metric. For example, we take e_{opt} as 10k, 30k, 10k, 15k, and 25k for the dataset Darmanis, Yan, Pollen, Klein, and Melanoma respectively.

Supplementary Note 4: Application of LSH-GAN on bulk RNA-seq data

We downloaded the bulk RNA-seq cortex data of WT (wild type) and AD (Alzheimer's disease) phenotype mice, which are known to form amyloid-beta (A) plagues, at three different ages (2M, 4M, and 7M) from a GSE104775 dataset released to the NCBI GEO website. We downloaded the processed data from Jinhee Park et al. [8], which consists of gene expression values of 1,208 DEGs over 36 samples. We utilized a random forest classifier for discriminating the real and generated samples by LSH-GAN (similar to the analysis given in the table-2 of the main text). The classifier gives a chance level performance (AUC score 0.57 ± 0.03 for 10 fold cv) for discriminating between real and generated samples, which suggests the generated samples are highly similar with real samples.

Supplementary References

[1] L. Pauleve, H. Jegou, L. Amsaleg, Locality sensitive hashing: A comparison of hash function types and querying mechanisms, Pattern Recognition Letters 31 (2010) 1348–1358.

[2] X.-L. Mao, B.-S. Feng, Y.-J. Hao, L. Nie, H. Huang, G. Wen, S2jsd-lsh: A locality-sensitive hashing schema for probability distributions, in: Proceedings of the AAAI Conference on Artificial Intelligence, volume 31, 2017.

[3] L. Yan, M. Yang, H. Guo, L. Yang, J. Wu, R. Li, P. Liu, Y. Lian, X. Zheng, J. Yan, et al., Single-cell rna-seq profiling of human preimplantation embryos and embryonic stem cells, Nature structural & molecular biology 20 (2013) 1131.

[4] A. M. Klein, L. Mazutis, I. Akartuna, N. Tallapragada, A. Veres, V. Li, L. Peshkin, D. A. Weitz, M. W. Kirschner, Droplet barcoding for single-cell transcriptomics applied to embryonic stem cells, Cell 161 (2015) 1187–1201.

[5] A. A. Pollen, T. J. Nowakowski, J. Shuga, X. Wang, A. A. Leyrat, J. H. Lui, N. Li, L. Szpankowski, B. Fowler, P. Chen, et al., Low-coverage single-cell mrna sequencing reveals cellular heterogeneity and activated signaling pathways in developing cerebral cortex, Nature biotechnology 32 (2014) 1053.

[6] S. Darmanis, S. A. Sloan, Y. Zhang, M. Enge, C. Caneda, L. M. Shuer, M. G. H. Gephart, B. A. Barres, S. R. Quake, A survey of human brain transcriptome diversity at the single cell level, Proceedings of the National Academy of Sciences 112 (2015) 7285–7290.

[7] I. Tirosh, B. Izar, S. M. Prakadan, M. H. Wadsworth, D. Treacy, J. J. Trombetta, A. Rotem, C. Rodman, C. Lian, G. Murphy, et al., Dissecting the multicellular ecosystem of metastatic melanoma by single-cell rna-seq, Science 352 (2016) 189–196.

[8] J. Park, H. Kim, J. Kim, M. Cheon, A practical application of generative adversarial networks for rna-seq analysis to predict the molecular progress of alzheimer's disease, PLoS computational biology 16 (2020) e1008099.
